# Case Report: Bilateral Palsy of the Vocal Cords After COVID-19 Infection

**DOI:** 10.3389/fneur.2021.619545

**Published:** 2021-05-19

**Authors:** Frederic Jungbauer, Roland Hülse, Fei Lu, Sonja Ludwig, Valentin Held, Nicole Rotter, Angela Schell

**Affiliations:** ^1^Department for Otorhinolaryngology, Head- and Neck-Surgery, University Medical Centre Mannheim, Mannheim, Germany; ^2^Department for Neurology, University Medical Centre Mannheim, Mannheim, Germany

**Keywords:** COVID-19, SARS-CoV-2, vocal cords, palsy, cranial nerve, recurrens

## Abstract

During the COVID-19 pandemic, adverse neurological effects have been described. In addition to unspecific neurological symptoms, cranial nerve deficits have appeared as part of SARS-CoV-2 infection. In this case report, we describe a 74-year-old patient who developed bilateral paralysis of the vocal cords some weeks following his dismissal in stable condition after COVID-19 pneumonia. After ruling out central lesions, peripheral tumors, and other possible causes, therapy was initiated with methylprednisolone, inhalations, and oxygen. The patient showed no improvement, so laterofixation after Lichtenberger was performed. The dyspnea worsened after several weeks, so a laser posterior cordectomy was performed with satisfactory outcome.

## Introduction

Severe Acute Respiratory Syndrome Corona Virus 2 (SARS-CoV-2) is a novel corona virus that was first detected in the Chinese city of Wuhan in 2019 (1). The resulting disease, Corona Virus Disease 2019 (COVID-19), has spread pandemically and is now a critical threat to the global healthcare system.

The pandemically significant pathomechanism leads to pneumonia with, in the most dramatic case, serious organ failure and respiratory decompensation, which leads to the need for ventilation. In addition, the majority of infections are a- or oligo-symptomatic, with the symptoms of a mild-to-moderate flu-like infection of the respiratory tract. In addition to cough and fever, sensory impairments, such as a reduction in smell and taste, are also observed (2). Besides these relatively harmless limitations, however, critical neurologic complications such as encephalitis and encephalomyelitis have been reported, especially in clinically severe cases (3).

In addition, affection of peripheral and cranial nerves in the context of a COVID-19 infection were also reported in several case reports and series, with paralysis of the eye muscles described most commonly (3).

Nevertheless, neuropathies of the facial nerve (4) and more caudally located nerves such as the phrenic nerve (5) have also been observed in COVID-19. The exact pathomechanisms are still unclear and in the interest of current research, a direct neurotoxic effect of the virus or a virally triggered autoimmune vasculitis of the nerves have been hypothesized (6).

In this context, the tenth cranial nerve, the vagus nerve, plays a special role. In addition to its sensitive, sensory and vegetative parts, it and its branches innervate various muscles in the head and neck region. The fibers innervating the larynx and the vocal cords also run over the vagus nerve and regulate the glottis, the narrowest and thus most critical point in the human airway. While unilateral vocal cord palsy mainly causes hoarseness, bilateral vocal cord palsy, especially in the acute situation, represents a dangerous respiratory emergency requiring immediate action. Neuropathy of the vagus nerve in the context of COVID-19 has already been described, at least partially, but with deficits in the more cranial pharyngeal region and simultaneous impairment of the ninth cranial nerve, the glossopharyngeal nerve (7).

This report describes the case of a patient with bilateral vocal cord palsy after undergoing COVID-19 infection, which could be a rare neuronal manifestation of SARS-CoV-2.

## Case Report

A 74-year-old female was admitted to our hospital after syncope, most likely of orthostatic genesis, during a bronchopulmonary infection and suspicion of COVID-19 pneumonia. Pre-existing conditions included high blood pressure, as well as implantation of knee endoprosthesis on both sides and hip endoprosthesis on the right side.

A computed tomography (CT) scan of the thorax showed frequently reported CT features of COVID-19 pneumonia and the nasopharyngeal smear for SARS-CoV-2 was PCR positive.

With the patient's consent, therapy with off-label use hydroxychloroquine was initiated. Due to persistent recurrent fevers, additional empirical antibiotic therapy with clarithromycin was initiated.

Six days after admission, the patient developed increasing respiratory insufficiency with deterioration of the peripheral SpO2, despite oxygen administration, and was urgently transferred to the intensive care unit. She was intubated and ventilated for a total of 17 days and was extubated successfully after that. The patient was then transferred to a peripheral hospital for early rehabilitation where she stayed for 9 days. At that time, she did not show any significant dyspnea.

Two weeks after discharge from the rehabilitation center, the patient was again urgently transferred to our hospital due to dyspnea. After acute cardiopulmonary reasons for the dyspnea were ruled out, an inspiratory stridor was noticed, and the patient was presented to the department of otorhinolaryngology, head and neck surgery as well as in our phoniatrics section.

**Figure 1:**
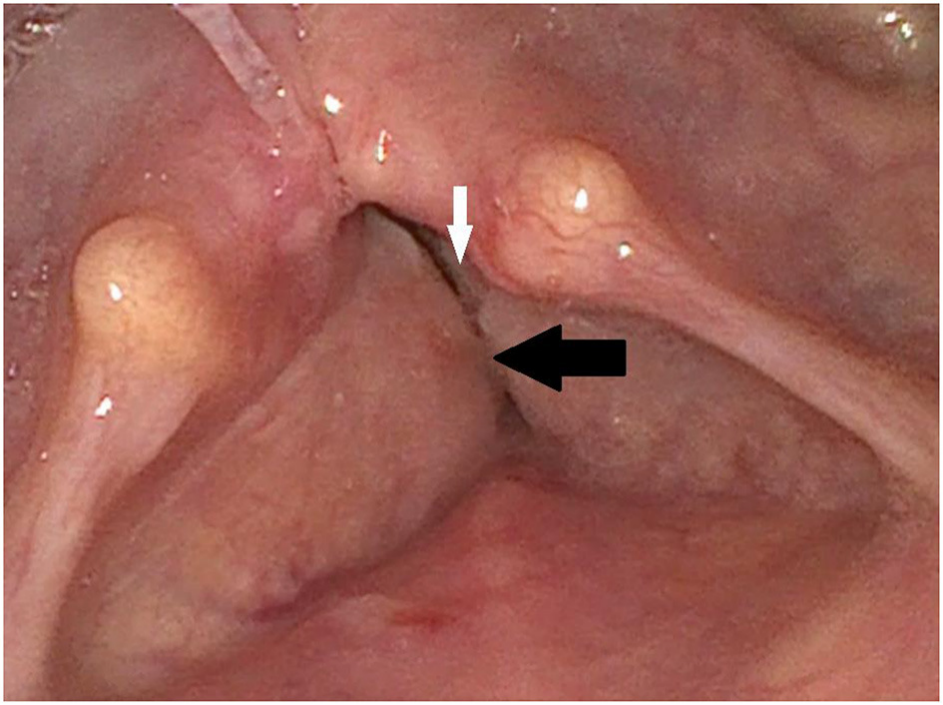
Initial finding via laryngoscopy: The paralyzed vocal cords (white arrow) are partially covered by the hyperplastic vestibular folds (black arrow).

Using the “RBH-scale,” the Roughness, Breathiness and Hoarseness of the speaking voice are each subjectively rated by the examiner as 0 (no disorder), 1 (low-grade disorder), 2 (moderate-grade disorder) or 3 (high-grade disorder). With the Voice Handicap Index (VHI), 3 domains (functional, physical, and emotional aspects of the voice disorder) are self-assessed by the patient with 10 questions each. Patients indicate whether the described stresses caused by the voice disorder occur in their everyday life never (0 points), almost never (1 points), sometimes (2 points), almost always (3 points), and always (4 points) (8). The patient showed a high graded dysphonia with a R3B1H3 in the RBH-*scale* and 67 points in the VHI. The voice sounded highly rough with pathological phonatoric effort. In the laryngostroboscopic examination, we found a bilateral vocal cord palsy with hyperplastic vestibular folds (Figure 1). During phonation we detected a small glottal gap with missing mucosal waves and amplitudes. Only some passive vibrations could be seen. During respiration the vocal folds were also fixed in medial to mediolateral position without recognizable lateral movements. During inspiration, the vocal folds were driven medially because of the Bernoulli effect. During pauses, it could be heard how the patient breathed heavily. The voice range profile was narrow with little dynamic range and no projection of voice.

Imaging via MRI and CT scan found no morphological cause for vocal cord palsy, no cerebral ischemia, bleeding, or cerebral or pulmonary tumor or other cervical or thoracic mass that could compress or affect recurrent laryngeal nerve function. Serological laboratory testing ruled out increased autoimmune parameters or an ongoing or past neurotropic viral infection. Furthermore, a neurological consultation showed no evidence of critical illness neuropathy or other signs and symptoms of neurological disease that could affect vocal cord function, e.g., progressive bulbar palsy. Consequently, it was assumed that the SARS-CoV-2 virus resulted in a neurological affection of the recurrent nerves.

We started a therapy with intravenous methylprednisolone, inhalations, and oxygen via nasal mask. While there was no improvement, we performed a laterofixation according to Lichtenberger (9) under intubation anesthesia, using a special endo-extralaryngeal needle carrier instrument to lateralize the paralyzed vocal fold in the sense of an arytenoidopexy. Apart from hyperplastic vestibular folds, which occur compensatory after vocal cord palsy, no morphologic features such as tumors or scars on the vocal cords were found intraoperatively.

**Figure 2:**
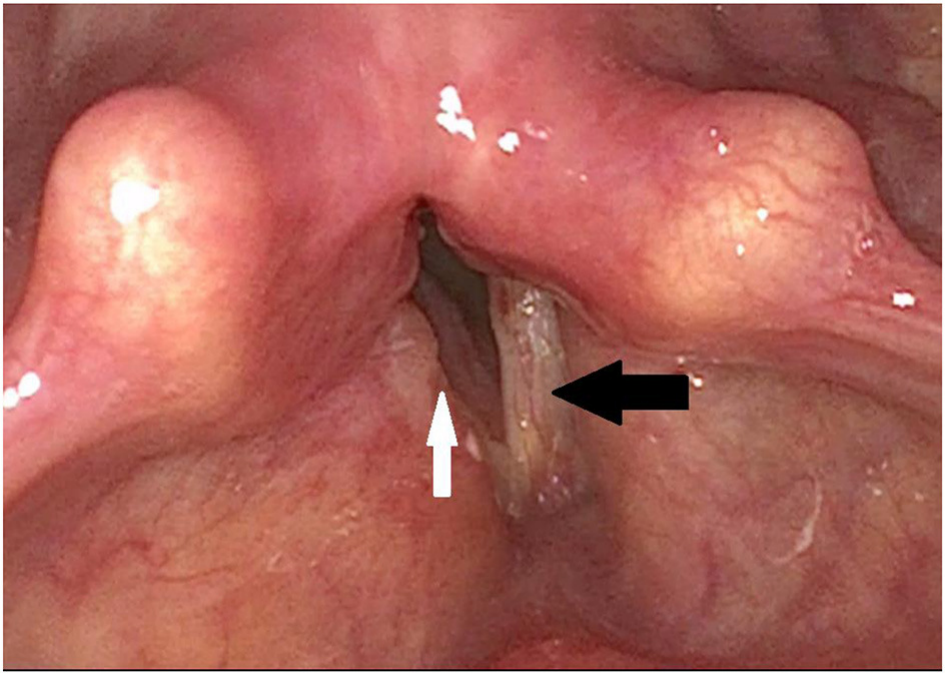
Post-operative finding via laryngoscopy after laterofixation on the right side: The right vocal fold is fixed laterally (white arrow) and thus widens the glottic gap, while the position of the left vocal fold is unchanged (black arrow). The vestibular folds are less swollen.

**Figure 3:**
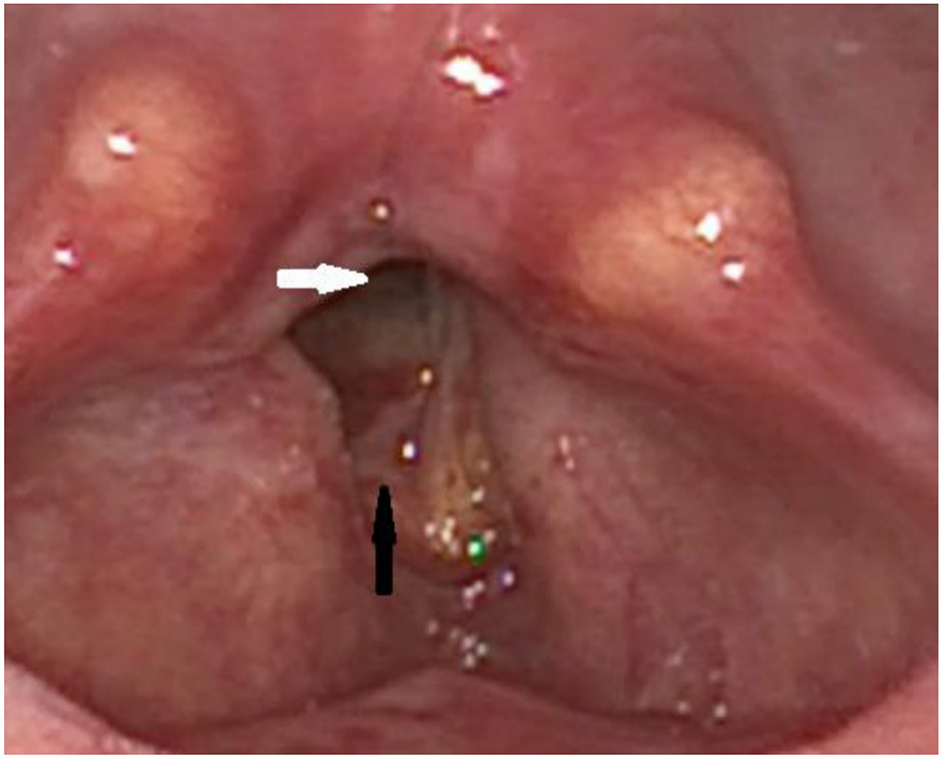
Post-operative finding via laryngoscopy after laser posterior cordectomy: The posterior portion of the glottic gap is open (white arrow), while the anterior portion of the right vocal fold is still swollen postoperatively (black arrow).

Thereafter, there was a marked improvement in dyspnea (Figure 2) and the patient could be discharged to outpatient follow-up care The patient still showed a high graded dysphonia that changed to a R1B3H3 in the “RBH”-*scale* and improved to 34 points in the VHI. The voice quality changed from a rough to a breathy voice due to the unmodulated air passing the post-therapeutic significant glottal gap. In the laryngostroboscopic examination, the fixed vocal folds hardly vibrated and there is a large glottal gap during attempted phonation. Still, there were no recognizable mucosal waves and amplitudes. The voice range profile was slightly improved, but there was still a high graded hoarseness as hearable in the additional material. After several weeks, the patient returned with worsening of dyspnea, and a laser posterior cordectomy was performed, which resolved the dyspnea, nevertheless it did not improve the patient's voice quality (Figure 3). The patient is currently stable and continues to be seen in regular intervals.

## Discussion

Different neurological symptoms have been described in the context of a SARS-CoV-2 infection.

In a systematic review, an overall average of 59.45% of patients experienced olfactory disturbance (3). Ageusia and anosmia are specific symptoms of COVID-19 and, in contrast to other flu-like infections of the upper airway, do not seem to be caused by nasal congestion or rhinorrhea (2). Rather, a neurotoxic effect of the virus on the sensory cells of the nasal and oral mucosa may occur (10). To determine whether the presented patient also showed sensory deficits, we performed olfactory testing. Hyposmia, (specifically identification, discrimination, and threshold) was found. Besides presbyacusis, other testing of the vestibular, gustatory, and hearing functions showed no signs of impairment. In light of the currently hypothesized pathomechanism of SARS-CoV-2 by binding to the angiotensin-converting enzyme 2 (ACE2) as his receptor and entering the nervous system (11), the evidence of anosmia suggests that the virus had infiltrated the patient's nervous system. This makes a neurotoxic effect of the virus on other nervous structures, such as the brain nerves, seem plausible, at least in theory. However, due to the fact that olfactory impairment seems to occur at a very high frequency in the context of COVID-19 infections, whereas motor paralysis is reported as a very rare phenomenon, no definite causal relationship can be deduced here either.

Besides a differential diagnosis of COVID-19 associated vocal cord palsy with the infection, we discussed intubation damage. However, there was no stridor during the inpatient stay in the rehabilitation center, and dyspnea was not pronounced at that time. In addition, intraoperatively there were no morphologic changes of the vocal folds (such as scarring strands) that would go beyond compensatory hyperplasia of the vestibular folds. Therefore, it seems unlikely that any bilateral intubation trauma would have become symptomatic and clinically noticeable only weeks later. However, no clear causal association between focal paralysis and infection with SARS-CoV-2 can be demonstrated at this stage of research.

The time from the classic symptoms of COVID-19, such as fever and cough, to the onset of neurologic failure was longer in our case, 46 days, than in most published cases of cranial nerve palsy. Specifically, it appears that the palsy of the eye muscles (controlled by the fourth, fifth and sixth cranial nerves) occurs earliest within the scope of the infection, namely within a few days (12–14). However, it should also be noted that there were still failures of the cranial nerves that control the eye muscles in individual cases with a significantly longer latency; e.g., bilateral trochlear palsy was only described after 11 days (15).

Palsy of the cranial nerves that lie further caudally, such as the seventh cranial nerve, has also been described after about 1 week, thus leading to COVID-associated Bells paralysis (4). Also branches of the cervical plexus like the phrenic nerve, which innervates the diaphragm, have also been described as the sites of COVID-associated paralysis. In these patients, specific symptoms appeared ~1 week after general symptoms of the disease began (5).

An affection of the ninth cranial nerve (the glossopharyngeal nerve) was described in a patient who developed oropharyngeal dysphagia 29 days after the first symptoms of COVID-19 (7). To the best of our knowledge, a report on paralysis of the 11th or 12th cranial nerves (accessory and hypoglossal nerves) has not yet been published.

Syndromes in which multiple cranial nerves fail, similar to Miller Fisher syndrome, are described with an onset latency of 3–5 days (12).

In summary, it can be speculated that cranial nerves, which are further cranial, are affected more often and show earlier failures, whereas caudal cranial nerves are affected less frequently, and if they are affected, they are affected later in the course of the disease. This observation is congruent with the fact that an affection of the brain stem was detected in a mouse model used in a previous study on the SARS-CoV virus and that this virus enters the brain primarily via the olfactory bulb (16).

The temporal differences in the involvement of the different can be determined by the anatomical location of the different nerves and their core areas; however, different theories exist on the pathophysiological mechanism of nerve damage. The virus appears to have a direct effect on the affected nervous structures, such as the olfactory bulb, when early-onset failures, such as odor reduction occur. For late-onset failures, the time course contradicts a direct neurotoxic effect of the virus because the late-onset palsy described above developed at times when the virus no longer caused systemic symptoms. Rather, it is currently assumed that molecular mimicry leads to a virally triggered autoimmune response of the infected person, which is directed against the nerves and their supplying vessels. This corresponds to the suspected pathomechanism in the post-viral olfactory dysfunction (17). Fotuhi et al. suggest a classification system called Neurocovid Stage I–III (6). In Neurocovid Stage I, the extent of SARS-CoV-2 binding to the angiotensin-converting-encyme-2 (ACE2) receptor is limited to the nasal and oral mucosa, resulting in a limitation in smell and taste. Persistence and progression of the infection can lead to Neurocovid Stage II where focal palsy occurs due to the above-mentioned molecular mimicry, possibly due to vasculitis in the nerves and muscles triggered by the hyperactivated immune system. In stage III, a cytokine storm damages the blood-brain barrier, including symptoms, such as delirium, encephalopathy and/or seizures.

Previous electron microscopic studies indicate that the virus has a high affinity for the ACE2 receptor and that the receptor plays an important role in penetrating the nervous system (18). Reports show that the incidence of hyposmia as a result of COVID-19 infection is different by region. While relatively few patients (5%) experienced hyposmia (19) in the initial infection area of Wuhan, China, European studies show significantly higher incidences of hyposmia (up to 88% of those infected) in the context of COVID-19 (20). Initial studies also show that a higher allele frequency of an intron variant of ACE2 (rs4646127) can be found in Asia (21) and that the different variants of the ACE2 receptor have a different affinity for SARS-CoV-2 (22). However, a clear risk gene for the neuronal manifestation of COVID-19 infection has yet to be identified. Therefore, it is speculated that therapy with ACE inhibitors might increase the risk of a nerve attack due to SARS-CoV-2. In our case, the patient was treated with an ACE inhibitor (ramipril) due to arterial hypertension. However, retrospective studies (23) do not seem to prove this and advocate for the continuation of drug therapy because of the organo-protective benefits of ACE inhibitors.

A retrospective study found neurological manifestations especially in patients with lymphopenia, thrombocytopenia, and higher blood-urea concentrations (19). Our case showed lymphopenia with 1.05^*^10E9/L (reference range 1.4–3.2^*^10E9/L) and thrombocytopenia with 117 ^*^ 10E9/L (reference range 165–387^*^10E9/L), but no increased urea values during the phase of nerve paralysis. However, during the initial COVID-19 pneumonia, there was an increased urea level with 108.3 mg/dl (reference range 21–43 mg/dl) and lymphocytopenia (1.04 ^*^ 10E9/L) with normal thrombocytes at the same time.

In most of the published case reports, therapy with i.v. corticoids and i.v. immunoglobulins were initiated, resulting in a clear improvement in symptoms overall. Recurrence palsy plays a special role in the case described in this report because its occurrence (especially bilateral) can lead to an acute life-threatening risk to the airway and spontaneous healing cannot occur.

## Conclusion

In addition to the typical symptoms and forms of manifestation, COVID-19 can cause complications in individual cases that do not correspond to the average appearance. Sensory and motor deficiency symptoms (a neuronal affection of SARS-CoV-2) should be considered, especially for high-risk patients, and further diagnostics and specific therapies should be initiated. Vocal cord paralysis can also occur as a rare complication in the setting of or following COVID-19 infection and may be due to the neurotoxic effects of SARS-CoV-2. It is important for clinicians to consider this as a possible differential diagnosis in COVID-19 patients with dyspnea. Close interdisciplinary coordination is also highly recommended.

## Data Availability Statement

The original contributions presented in the study are included in the article/Supplementary Material, further inquiries can be directed to the corresponding author/s.

## Ethics Statement

Written informed consent was obtained from the individual(s) for the publication of any potentially identifiable images or data included in this article.

## Author Contributions

FJ did the literature research and wrote the manuscprit. AS initiated the writing of the manuscript, coordinated the different disciplines involved in the case and reviewed the manuscript with overall expertise and in terms of language. RH and FL performed the clinical examination, documentation, creation of the additional material, and reviewed the manuscript with regard to phoniatric expertise. NR and SL reviewed the manuscript with regard to expertise in otorhinolaryngology, head- and neck-surgery. VH reviewed the manuscript with regard to neurologic expertise. All authors contributed to the article and approved the submitted version.

## Conflict of Interest

The authors declare that the research was conducted in the absence of any commercial or financial relationships that could be construed as a potential conflict of interest.
